# The double-edged effects of IL-6 in liver regeneration, aging, inflammation, and diseases

**DOI:** 10.1186/s40164-024-00527-1

**Published:** 2024-06-18

**Authors:** Min-Jun Wang, Hai-Ling Zhang, Fei Chen, Xiao-Jing Guo, Qing-Gui Liu, Jin Hou

**Affiliations:** 1 Department of Cell Biology, Center for Stem Cell and Medicine, Second Military Medical University (Naval Medical University), Shanghai, China; 2 National Key Laboratory of Immunity and Inflammation, Institute of Immunology, Second Military Medical University (Naval Medical University), Shanghai, China; 3 Department of Neurology, Changhai Hospital, Second Military Medical University (Naval Medical University), Shanghai, China; 4 Department of Health Statistics, Faculty of Health Service, Second Military Medical University (Naval Medical University), Shanghai, China

**Keywords:** IL-6, Liver injury, Fibrosis, steatosis, carcinogenesis

## Abstract

Interleukin-6 (IL-6) is a pleiotropic cytokine and exerts its complex biological functions mainly through three different signal modes, called *cis-*, *trans-*, and cluster signaling. When IL-6 binds to its membrane or soluble receptors, the co-receptor gp130 is activated to initiate downstream signaling and induce the expression of target genes. In the liver, IL-6 can perform its anti-inflammatory activities to promote hepatocyte reprogramming and liver regeneration. On the contrary, IL-6 also exerts the pro-inflammatory functions to induce liver aging, fibrosis, steatosis, and carcinogenesis. However, understanding the roles and underlying mechanisms of IL-6 in liver physiological and pathological processes is still an ongoing process. So far, therapeutic agents against IL‑6, IL‑6 receptor (IL‑6R), IL-6-sIL-6R complex, or IL-6 downstream signal transducers have been developed, and determined to be effective in the intervention of inflammatory diseases and cancers. In this review, we summarized and highlighted the understanding of the double-edged effects of IL-6 in liver homeostasis, aging, inflammation, and chronic diseases, for better shifting the “negative” functions of IL-6 to the “beneficial” actions, and further discussed the potential therapeutic effects of targeting IL-6 signaling in the clinics.

## Introduction

Interleukin-6 (IL-6) is a small glycoprotein composed of 184 amino acids. Its molecular weight is 21–28 kDa with a four-helix bundle structure, the characteristic of the IL-6 cytokine family [1]. IL-6 is considered as a pleiotropic cytokine for its multiple physio-pathologic functions. Under normal conditions, the levels of IL-6 in the blood and interstitial fluid are extremely low. During aging, inflammation, or other pathological conditions, especially in the liver, IL-6 levels are significantly increased and are crucial for the progression of inflammation, fibrosis and carcinogenesis [1, 2]. However, the deletion of *il6* gene also impairs the hepatocyte homeostasis and liver regeneration [3–5]. Understanding the roles and underlying mechanisms of IL-6 in liver physiological and pathological processes is still an ongoing process, and is critical to the development of therapeutic strategies for liver diseases.

### IL-6 and its effector signaling

**Fig. 1:**
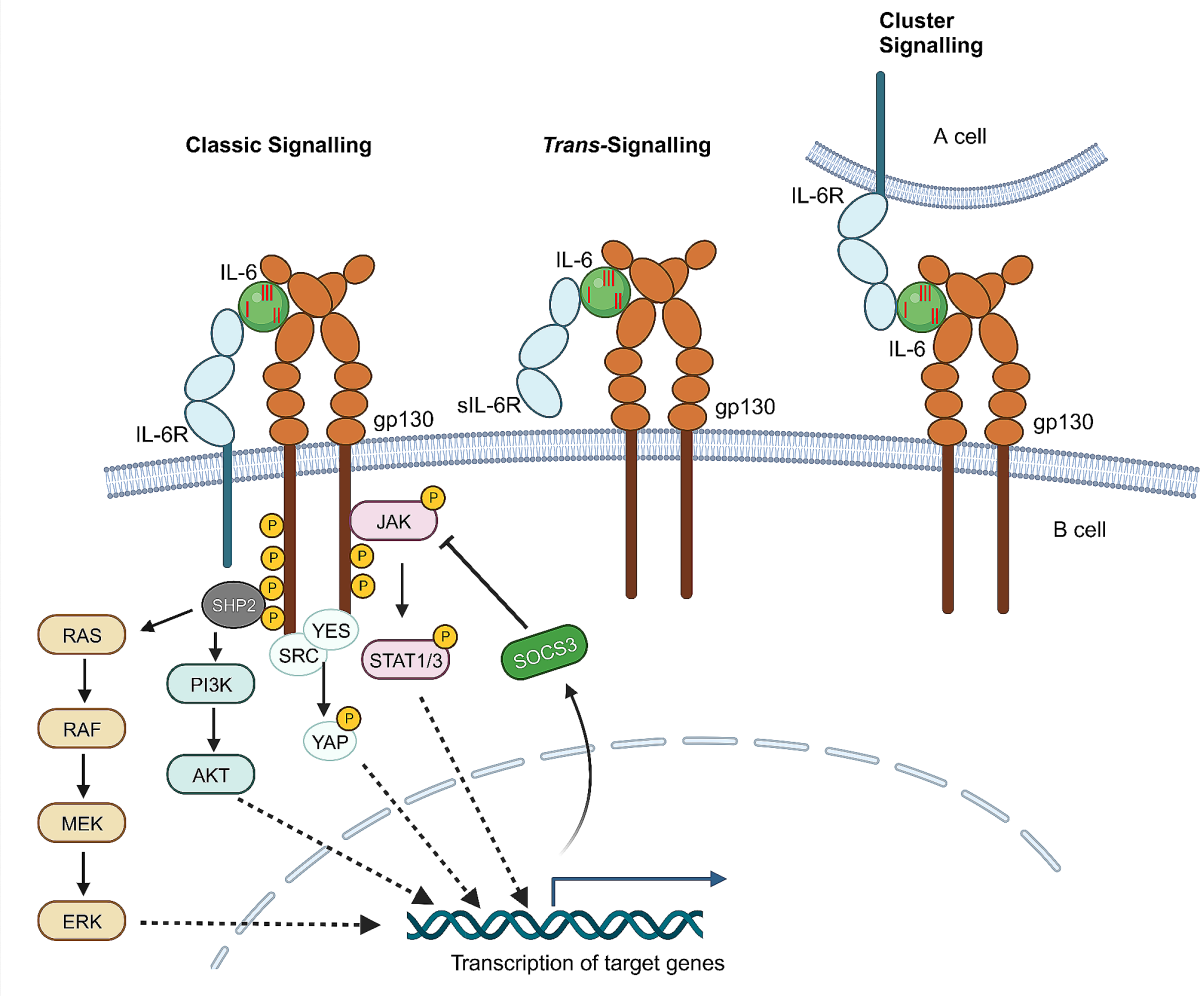
Modes of IL-6 signaling and the intracellular signal transduction. The *cis-*, *trans-*, and cluster signaling of IL-6 complex. With the activation of IL-6 signaling complex and the phosphorylation of gp130 intracellular domain, JAK-STAT, MAPK, PI3K, and YAP pathways are activated to initiate the transcription of target genes. SOCS3 is a direct target of STAT3 and provides negative feedback to suppress JAK-STAT3 signaling

The effector signaling of IL-6 contains three modes (Fig. 1) [6]. IL-6 receptor (IL-6R) is a specialized receptor for IL-6, which is located on the membrane of a set of cell types, such as hepatocytes, immune cells, and some endothelial cells. In addition to IL-6R, IL-6 effector signaling needs another receptor component, 130-kD glycoprotein (gp130) protein. The first signal mode is called classic signaling, also named *cis*-signaling. The conserved site I of IL-6 cytokine first binds to the membrane bound IL-6R, and then the conserved sites II and III of IL-6 acquire the capability to interact with gp130 and form the complex (Fig. 1) [2], which leads to the dimerization and activation of gp130. The association between the conserved site I of IL-6 and IL-6R is indispensable, as the conserved sites II and III of IL-6 alone are not able to bind gp130 receptor and initiate its dimerization. The second signal mode is IL-6 *trans*-signaling, for that IL-6 conserved site I first binds a soluble IL-6R (sIL-6R), and then conserved sites II and III bind gp130 on a nearby cell to induce its dimerization and activation (Fig. 1) [7]. For the soluble IL-6 receptor, it is from the membrane bound IL-6R, which is cleaved at the cell surface by A Disintegrin and Metalloprotease17 (ADAM17) and released to tissue interstitial fluid and blood [8]. The third signal mode is cluster signaling. In this mode, IL-6 conserved site I binds a membrane bound IL-6R in A cell, and then the conserved sites II and III within IL-6/IL-6R complex bind gp130 located on the membrane of B cell. The A cell operates as a transmitter cell to activate gp130 downstream signaling of B cell, which is recently described in the dendritic cells (DCs)-mediated activation of IL-6 effector signaling in T cells (Fig. 1) [9, 10]. Collectively, IL-6 *trans*-signaling and cluster signaling modes can occur in the cells which only express gp130 but not IL-6R, thus amplifying the spectrum of target cells in response to IL-6.

Upon the formation of IL-6, IL-6R, and dimerized gp130 complex, the intracellular domain of gp130 is phosphorylated. The phosphorylated gp130 then recruits the downstream signal transducers, resulting in the activation of several signaling pathways (Fig. 1) [2, 11]. Janus kinase (JAK)-Signal Transducer and Activator of Transcription (STAT) signaling is the well-established cascade downstream of activated gp130. STAT1 and STAT3 are phosphorylated by JAKs and then form active dimers (homo- and hetero-dimers), which translocate into the nucleus to induce the transcription of IL-6 target genes [12]. The activated gp130 also recruits Src homology 2 domain-containing protein tyrosine phosphatase 2 (SHP2), leading to the activation of downstream extracellular-signal regulated kinase (ERK)/mitogen-activated protein kinase (MAPK) and phosphatidylinositol 3-kinase (PI3K)/AKT pathways. Additionally, YES-associated protein 1 (YAP) and Src family kinase (SRC) are activated by phosphorylated gp130, resulting in YAP phosphorylation and nuclear translocation to initiate downstream transcription [13]. Furthermore, the activation of IL-6 effector signaling can also lead to a negative feedback regulatory system. For example, the expression of suppressor of cytokine signaling 3 (SOCS3) is induced by the activated STAT3, and then binds the phosphorylated domains of gp130 and JAKs to induce their degradation [14]. Based on the three signal modes to activate gp130 and a set of downstream signaling cascades, IL-6 performs its multifaceted functions in tissue homeostasis, regeneration, aging, and inflammation, thus playing critical roles in the physiological and pathological processes.

### IL-6 in liver regeneration

**Fig. 2:**
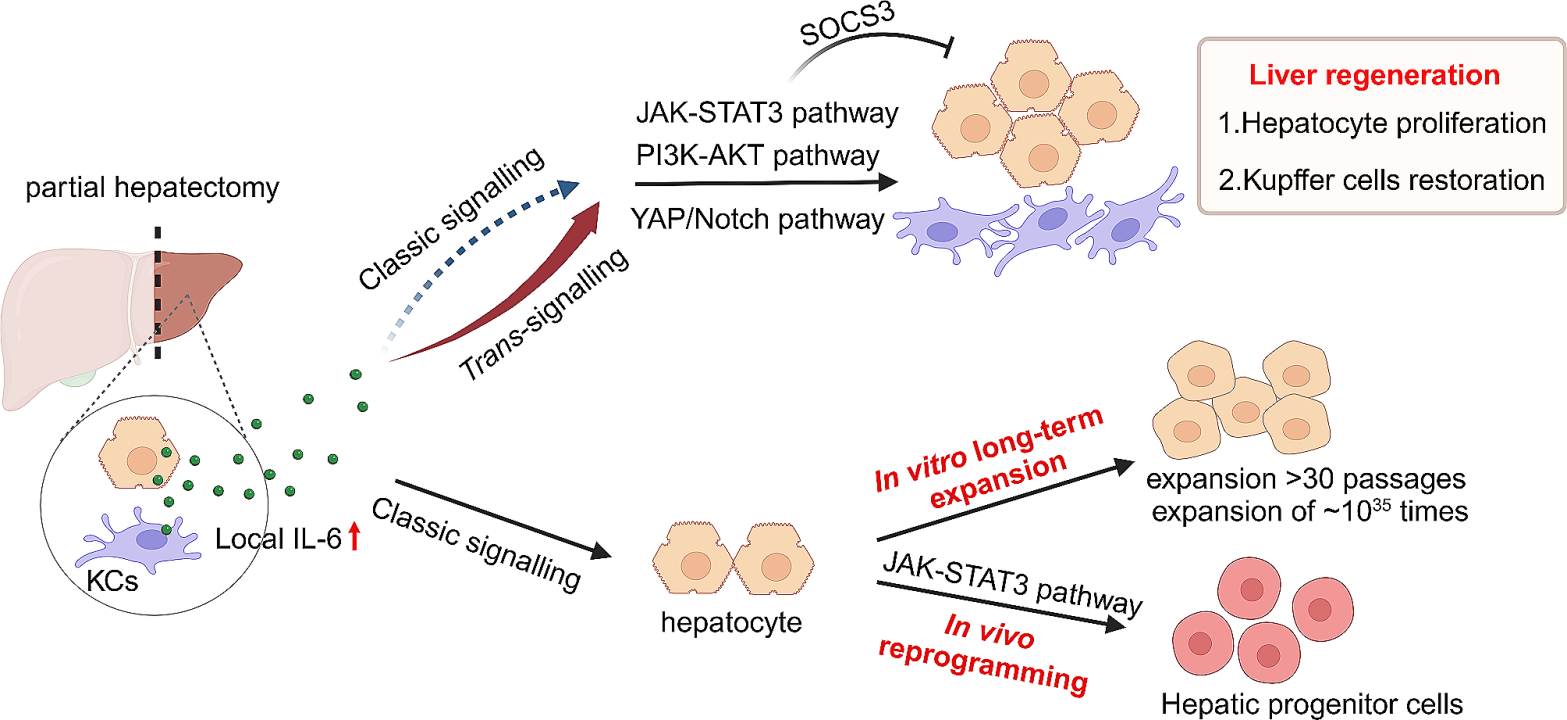
The role of IL-6 signaling in liver homeostasis and regeneration. IL-6 trans-signaling, not classic signaling, is more important for its contribution to liver regeneration. IL-6 classic signaling is significant for the in vitro long-term expansion of hepatocytes and the in vivo reprogramming of hepatocytes into hepatic progenitor cells

The liver possesses remarkable regeneration capacity under injury conditions. The critical role of the IL-6/IL-6R/gp130-JAK-STAT3 axis has been well-established in the initiation phase of liver regeneration after partial hepatectomy (Fig. 2) [15, 16]. IL-6 is rapidly produced by Kupffer cells, endothelial cells, and hepatocytes after partial hepatectomy, contributing to the following hepatocyte proliferation and parenchyma restoration. The IL-6-promoted liver regeneration was determined in IL-6 knockout mice, as the deletion of *il6* impaired the compensatory proliferation of hepatocytes by reducing the downstream STAT3 activation [3, 4], thus promoting liver failure, which could be corrected by the treatment of IL-6. This phenomenon and conclusion have been confirmed in hepatocyte-specific gp130-knockout, hepatocyte-specific STAT3-knockout, or IL-6R deficient mice [17–19]. IL-6 can also activate PI3K/AKT and YAP/Notch pathways independent of the STAT3 pathway to promote hepatocyte compensatory proliferation and liver regeneration [13, 20]. On the other side, the deletion of the negative regulator SOCS3 in hepatocytes resulted in the increased compensatory proliferation and more rapid restoration of liver mass following partial hepatectomy [21]. In addition, except produced by hepatocytes, IL-6 is also produced by myeloid cells, both of them are necessary to restore the number of liver-resident Kupffer cells to maintain tissue homeostasis during liver regeneration [22]. Other sources, including systemic IL-6 or skeletal muscle-derived IL-6, move into the liver and activate its effector signaling to trigger the autophagic flux in hepatocytes, which then contributes to liver regeneration [23, 24].

Although the classic or *cis*-signaling of IL-6 is determined to promote the compensatory proliferation of hepatocytes, its *trans-*signaling in liver regeneration is more important [19, 25–27]. During liver regeneration after 70% hepatectomy, hyper-IL-6, which is a fusion protein of IL-6 and sIL-6R, induced a stronger and a much earlier hepatocyte regeneration effect [25]. Similarly, following D-gal-induced liver damage, the administration of hyper-IL-6 for the activation of IL-6 *trans*-signaling could rescue animals through an enhanced hepatocyte regeneration [26]. IL-6/sIL-6R double transgenic mice showed massive hepatocyte proliferation even without hepatectomy, whereas IL-6 single transgenic mice did not show such a phenotype [28]. Moreover, it is reported that IL-6 *trans*-signaling not just accelerate liver regeneration, it is indispensable for the regeneration of liver after partial hepatectomy [19]. This work demonstrated that, during liver regeneration after 70% hepatectomy, sIL-6R can be expressed by not only hepatocytes, but also neutrophils and macrophages, and sIL-6R-mediated IL-6 *trans*-signaling forms the long-term contribution of IL-6 effects for liver regeneration [19]. Moreover, IL-6 classic signaling is activated in the acute phase-response post liver infection, however, the expression of acute phase protein serum amyloid A2 is similar in wild-type and sIL-6R^+/+^ (loss of membrane IL-6R) mice, explaining that IL-6 *trans*-signaling can completely compensate for the loss of IL-6 classic signaling [19]. Indeed, hepatocytes express far more amounts of gp130 on the surface than IL-6R, and hyper-IL-6 thus can lead to a profound downstream effector signaling [5, 27, 29]. Since IL-6 is rapidly internalized whereas the IL-6/sIL-6R complex is not, the IL-6 *trans*-signal therefore acts much longer [28]. Thus, the clinic use of hyper-IL-6 would be more effective to promote liver regeneration in patients post partial liver resection.

It is incontrovertible that hepatocytes have amazing proliferative capacity in liver regeneration and IL-6 plays a critical role during this process [30]. However, hepatocytes are very difficult to culture or expand in vitro, greatly limiting their clinical application in the therapy of liver diseases. It is reported that IL-6/sIL-6R combined with hepatocyte growth factors (HGF) significantly promoted hepatocyte proliferation after partial hepatectomy [20]. Similarly, a recent study reported that IL-6, co-operated with epidermal growth factor (EGF) and HGF, could promote the long-term expansion (> 30 passages in ~ 150 days with theoretical expansion of ~ 10^35^ times) of primary mouse hepatocytes in vitro (Fig. 2) [31], which may effectively resolve the limited application of hepatocyte transplantation in liver diseases due to a shortage of enough hepatocytes. In addition, liver progenitor cells are the alternative cell resource to repair the injured liver via their differentiation into hepatocytes [32]. Yet, the activation of liver progenitor cells during liver injury is somewhat restrained, leading to the insufficient contribution to the repairment of liver function. Recent studies suggest that IL-6 is highly expressed and secreted by liver-resident macrophages upon liver damage, and the produced IL-6, as a niche signal, binds the membrane IL-6R and gp130 of hepatocytes and then activates the downstream transcription factor STAT3. Phosphorylated STAT3 associates with the regulatory genomic regions of reprogramming- and progenitor-related genes, which in-turn reprograms the remaining hepatocytes into a progenitor-like state [32–35]. It is unexpected that a single IL-6 cytokine would possess the prominent ability to induce the highly efficient in vivo reprogramming of hepatocytes (Fig. 2). These findings also provide further evidences for that hyper-IL-6 is able to initiate the proliferation of liver progenitor cells in vivo to regenerate the impaired liver [36].

### IL-6 in liver aging

**Fig. 3:**
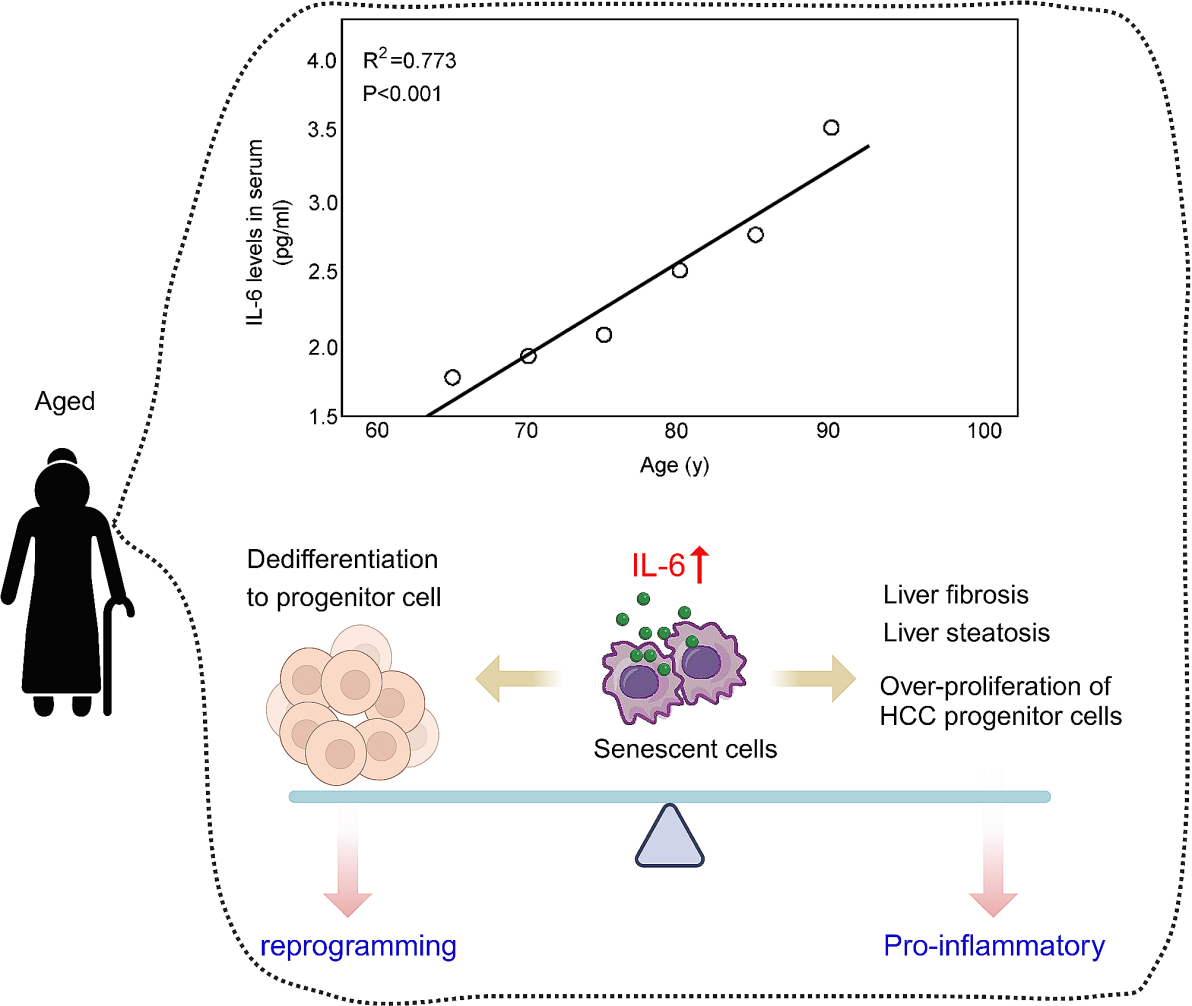
The role of IL-6 in liver aging. IL-6 concentration in serum is positively- corrected with the increased aging. Increased IL-6 in the liver is mainly expressed and secreted by senescent cells including hepatocytes, macrophages, and endothelial cells, leading to the development of aging-associated chronic liver diseases. On the other side, senescence-secreted high IL-6 can stimulate the reprogramming of cells by the paracrine signaling

Senescent cells are characterized by the expression of senescence-associated secretory phenotype (SASP) [37, 38], which promotes the development of various chronic diseases. IL-6, one of the major SASP factors, also belongs to the pro-inflammatory cytokines [38]. In the serum of healthy adults, IL-6 is normally lower than 2 pg/mL or undetectable, while a gradual increment of serum IL-6 amounts with advancing ages (Fig. 3) [39, 40]. Moreover, numbers of studies support the potential correlation of IL-6 levels with aging and chronic morbidity [38, 41]. As the data shown in the cohort of end-stage liver diseases, elevated IL-6 levels were highly predictive for mortality. None of the patients with lower IL-6 level (< 5.3 pg/mL) died within one year, but more than half of aged patients died within one year under higher IL-6 level (> 11.6 pg/mL) [41]. When exposed to hepatic ischemia/reperfusion insult, aged liver with high IL-6 in the microenvironment aggravates liver injury such as the intrahepatic tissue damage and inflammation [42]. Moreover, activation of IL-6 effector signaling is positively correlated with age-related dysregulation of lipid metabolism, hepatitis, fibrosis, and xenobiotic detoxification [1, 43]. During the senescence of liver cells, including hepatocytes, cholangiocytes, and stellate cells, IL-6 is produced and participate in the development of liver diseases, especially cancer carcinogenesis and progression [44, 45]. During carcinogenesis, senescence-related IL-6 activates gp130-STAT3 pathway using *trans*-signaling mode in hepatic progenitor cells or HCC progenitor cells to promote their proliferation, malignant transformation, and hepatocellular-cholangiocarcinoma carcinogenesis. On the other side, IL-6 expressed by senescent cells suppresses the proliferation of senescent hepatocytes, and the hepatic progenitor cells enter compensatory proliferation, thus further promoting hepatocarcinogenesis [44, 45]. Blocking IL-6 *trans*-signaling using soluble gp130 or clearing senescent cells using a senolytic agent, as well as the depletion of hepatic progenitor cells, result in a significant reduction of hepatocellular-cholangiocarcinoma tumors [44, 45]. As mentioned in the regeneration section, local IL-6 reprograms hepatocytes into hepatic progenitor cells via gp130-STAT3 pathway, and then promotes liver regeneration [33]. However, in chronic senescent conditions, IL-6 is produced by senescent cells to promote the over-proliferation of hepatic progenitor cells and contribute to hepatocellular-cholangiocarcinoma carcinogenesis. A recent study also found that hepatic stellate cells (HSCs) underwent senescence after partial hepatectomy, and elimination of these senescent HSCs impaired liver regeneration. The underlying mechanism was that senescent HSCs expressed and secreted IL-6, binding to membrane IL-6R of hepatocytes to stimulate liver regeneration by the STAT3 pathway and SRC/YAP signal activation [46]. In the aged liver, IL-6 can be expressed and secreted by all senescent liver cells, implying its multifaced actions in the development of age-related diseases (Fig. 3).

Interestingly, IL-6 also plays different roles in the activation of stem cells during aging. When in young mice upon damage, IL-6 is up-regulated and promotes the activation of gland’s stem cells in vivo and in vitro, which is determined in stem cell-derived organoids. However, local IL-6 level is elevated in the aging gland, which does not generate a pituitary stem cell activation but promotes inflammation [47]. Four transcriptional factors abbreviated as OSKM (Oct4, Sox2, Klf4, and c-Myc) are well-established to induce the dedifferentiation and cellular reprogramming in multiple tissues [48]. Actually, OSKM induces two opposite cellular fates, both reprogramming and senescence [49]. However, the OSKM-induced senescence also produces IL-6 to mediate the dedifferentiation and reprogramming, further suggesting the double-edged effects of IL-6 in rejuvenation and aging (Fig. 3) [50].

### IL-6 in liver diseases

**Fig. 4:**
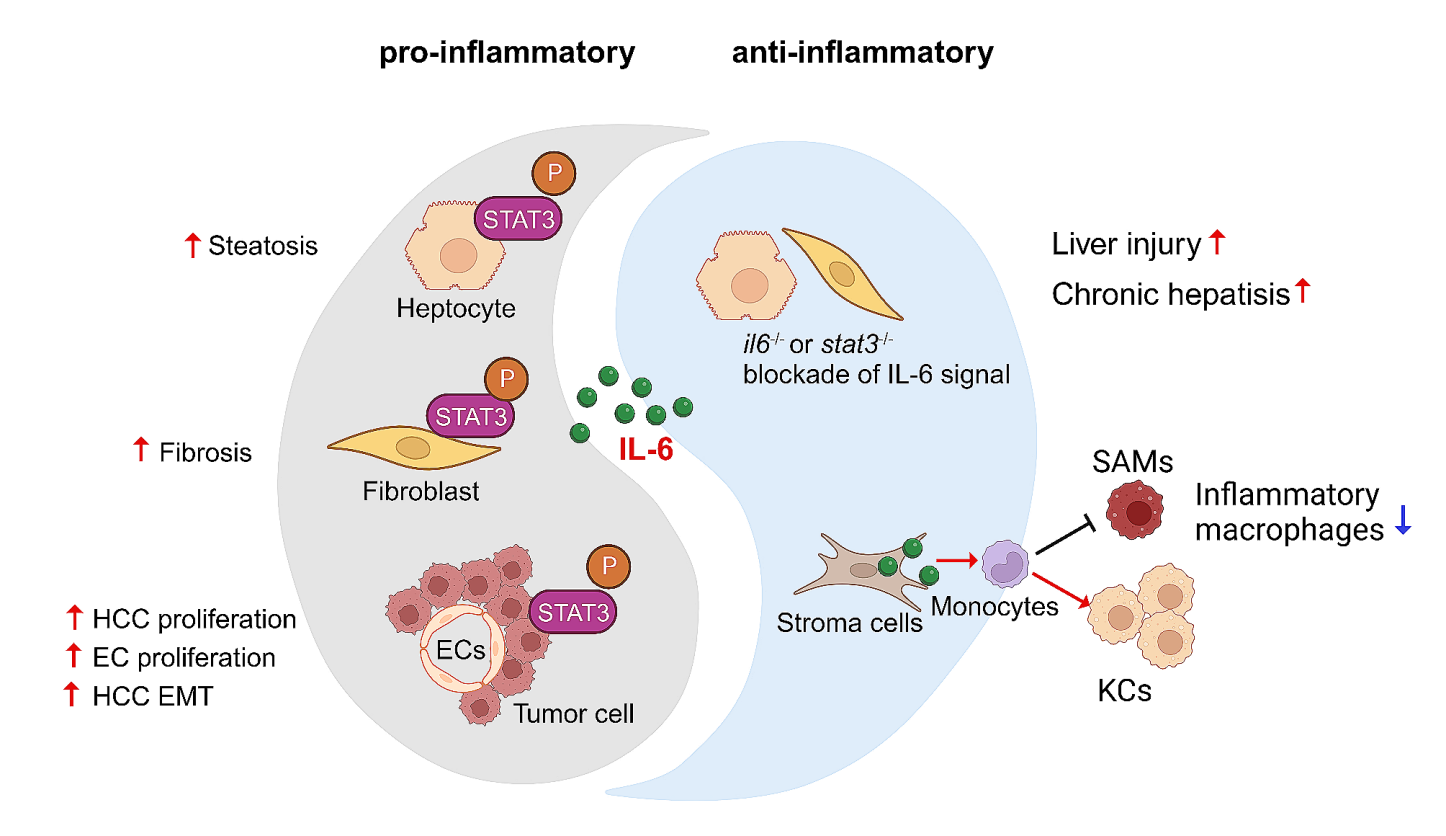
The pro-inflammatory and anti-inflammatory effects of IL-6 in the liver pathological process. The overexpression of IL-6 induced by liver injury presents a double-edged sword effect on the hepatic pathological process. Its pro-inflammatory effect promotes liver steatosis, fibrosis, endothelial cells (ECs) proliferation, and HCC development. On the other side, liver injury or chronic hepatitis are promoted in IL-6 or STAT3 knockout mice, suggesting the anti-inflammatory effect of IL-6. Moreover, IL-6 secreted by liver stroma cells can induce the differentiation of recruited monocytes towards tissue-resident Kupffer cells, but away from SAMs, so as to limit hepatic inflammation

IL-6 signal, also considered as a double-edged sword, performs both pro-inflammatory and anti-proinflammatory roles in the pathogenesis of liver diseases (Fig. 4). During inflammation, the hepatic IL-6 levels can be increased to more than 100 ng/mL [51]. For its pro-inflammatory effect, IL-6 classic signaling especially hepatocyte-specific gp130 activation is crucial for the induction of the acute phase proteins in the liver during host response to the infectious insults [52, 53], and this pro-inflammatory circumstance is quickly resolved when the infection is controlled. If repeated or chronic hepatic IL-6 inflammatory insults are raised, a set of liver diseases will be initiated. Many studies have highlighted that chronic exposure to IL-6 impaired hepatic lipid metabolism [54–56]. In the humanized liver mouse model, the inflammatory effect of IL-6/GP130 pathway promoted hepatic lipid accumulation, suggesting the therapeutic potential of antagonizing GP130 signaling in the treatment of liver steatosis [57]. A recent study used hPSC-derived liver culture to mimic genetic variant-derived NAFLD, and found that IL-6 expression and IL-6/STAT3 activity was elevated during NAFLD development. The dampening of IL-6/STAT3 activity could alleviate the genetic variant-mediated susceptibility to NAFLD [58]. In a mouse model of non-alcoholic steatohepatitis (NASH), knockout of IL-6 or IL-6R also reduced the signs of inflammation during NASH progression [59]. In severe COVID-19 patients, high level of IL-6 was produced in lung and acted on liver sinusoidal endothelial cells by IL-6 *trans*-signaling, inducing liver inflammation and even liver injury [60]. In the event of hepatocarcinogenesis and progression, increased serum IL-6 level and activated IL-6/STAT3 signaling is highly associated with early tumor recurrence and poor prognosis of HCC patients [61]. For example, inhibition of IL-6 signaling dramatically impedes tumorigenesis and extends the tumor-free survival of patients following surgical partial hepatectomy [62]. Indeed, IL-6 or gp130 knockout mice developed significantly less tumors in the diethylnitrosamine (DEN)-induced HCC model and prolonged survival [63, 64], and we found that the HCC progenitors HcPCs not only autocrined IL-6, but also with promoted responses to IL-6, for their malignant progression to established HCC [65, 66]. In intrahepatic cholangiocarcinoma (ICC), IL-6 increases the expression of circRNA GGNBP2 to encode the protein cGGNBP2-184aa, which in turn forms a positive feedback loop of IL-6/cGGNBP2-184aa/STAT3 to facilitate ICC progression [67]. In addition, HCC-derived fibroblasts can secrete IL-6 and bind adjacent HCC cells to activate IL-6/IL-6R/STAT3 axis, which facilitates epithelial-mesenchymal transition (EMT) of HCC cells and accelerated HCC development [68]. Moreover, increased IL-6 signal with impaired degradation of IL-6 cytokine family signal transducers promotes the proliferation and migration of HCC cells [69]. IL-6 also activates hepatocytes to produce serum amyloid A1 and A2, and forms the pro-metastatic niche to help the metastasis of pancreatic and colorectal cancer cells into the liver [70]. In addition, evidences suggest that IL-6 *trans*-signaling, but not IL-6 classic signaling, is essential to promote HCC carcinogenesis and progression, and only the activation of membrane-bound IL-6R and gp130 in hepatocytes seems not sufficient for tumor formation [71, 72]. Only the specific inhibition of IL-6 *trans*-signaling, rather than total inhibition of IL-6 signaling, is sufficient to suppress tumor progression [71, 72]. Together, the pro-inflammatory roles of IL-6 promotes the progression of chronic liver diseases, such as NAFLD, NASH, and HCC.

For the anti-inflammatory effect of IL-6, a set of studies in animal models involving chronic hepatitis demonstrate that IL-6 signaling is crucial for ameliorating liver injury and fibrosis. For example, *Il6*^−/−^ mice developed mature-onset obesity and decreased glucose tolerance, which was partly reversed by IL-6 replacement at low doses [73]. In the *mdr2*^*−/−*^ mouse model, the deletion of *il6* gene or blockade of IL-6 *trans*-signaling by the soluble gp130 form exacerbated hepatic steatosis, inflammation, chronic hepatitis, and hepatocarcinogenesis, suggesting the protective role of IL-6 signaling [74]. IL-6 *trans*-signaling has also been found to have protective roles on acetaminophen-induced acute liver injury in the mice model [75]. It is also reported that high-fat diet (HFD) caused more body-weight gain in *il6*^−/−^ mice than in wild-type mice [59]. HFD also exaggerated insulin resistance and deterioration of glucose homeostasis in *Il6ra*^Δmyel^ mice, with the underlying mechanism that specific inhibition of IL-6R expression resulted in M1 macrophage polarization and inflammation [76]. For macrophages in the liver, they are divided into the liver-resident Kupffer cells and monocyte-infiltrated macrophages. Tissue resident Kupffer cells’ main function is to maintain homeostasis. Monocyte-infiltrated macrophages are from the differentiation of recruited monocytes when exposed to liver injury [77]. In recent studies, it was found that human liver stromal cells produced and secreted IL-6 cytokine, contributing to the skewing differentiation of monocytes towards tissue-resident Kupffer cells but away from scar-associated macrophages (SAMs) (Fig. 4). Additionally, local IL-6 level is decreased in early-stage human liver diseases as compared to healthy liver tissues, suggesting a protective role for local IL-6 in the healthy liver [78].

### Therapeutic strategy of blocking IL-6 signaling

**Fig. 5:**
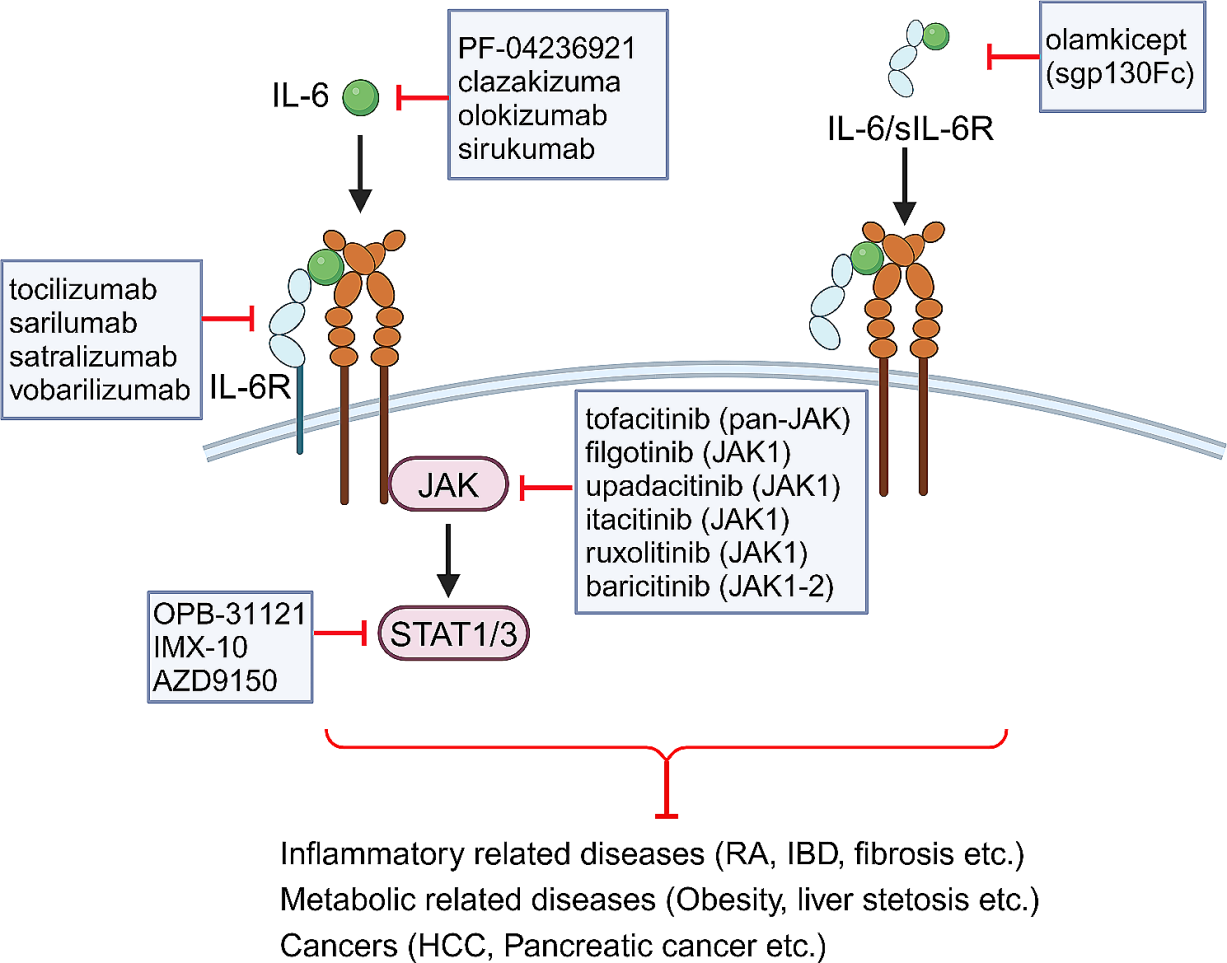
Strategies to specifically block IL-6 signaling. IL-6 signaling can be selectively blocked by antibodies or small molecules targeting the IL-6, IL-6R, sIL-6R, as well as intercellular downstream molecules JAKs and STATs. These neutralizing antibodies or small molecules against IL-6 signaling have been approved for the clinical treatment of inflammatory diseases, metabolic diseases, and cancers

As IL-6 classic and *trans*-signaling are involved in the development of various diseases, several strategies have been used to inhibit IL-6 effector signaling at different levels in the pre-clinical and clinical settings (Fig. 5; Table 1) [1, 2, 79]. Neutralization of IL-6 inhibits both classic and *trans*-signaling, and targeting IL-6R blocks all the three modes of IL-6 effects. Moreover, the use of sgp130Fc, targeting IL-6 and sIL-6R complex, selectively suppresses the *trans*-signaling of IL-6. As IL-6 classic signaling plays crucial roles in the initiation of inflammation and the host defense against pathogen infection, global blockade of IL-6 signaling may reduce the induction of hepatic acute-phase proteins upon infection, such as *Listeria monocytogenes* [79]. However, the specific blockade of IL-6 *trans*-signaling using the recombinant sgp130Fc did not interfere with its functions in the defense against pathogens [80].

**Table 1:** Clinical trials for the effects of IL-6 inhibition

Target	Drugs	Diseases	Clinical trials
IL-6	PF-04236921	Crohn’s disease	Phase II (NCT01345318)
	olokizumab	Rheumatoid arthritis Crohn’s disease	Phase III (NCT03120949) Phase II (NCT01635621)
	siltuximab	Pancreatic cancer Rheumatoid arthritis	Phase I (NCT04191421) Phase I (NCT02404558)
	clazakizumab	COVID-19 Crohn’s disease	Phase II (NCT04348500) Phase II (NCT01545050)
IL-6R	tocilizumab	rheumatoid arthritis COVID-19 Systemic Sclerosis Castleman’s disease	Phase IV (NCT01331837) Phase III (NCT04356937) Phase III (NCT02453256) Phase II (NCT01441063)
	sarilumab	COVID-19 Rheumatoid arthritis	Phase III (NCT04327388) Phase III (NCT02121210)
	satralizumab	Neuromyelitis Optica	Phase III (NCT02028884)
sIL-6R	olamkicept	Active ulcerative colitis	Phase II (NCT03235752)
Pan-JAK	tofacitinib	Ulcerative colitis Rheumatoid arthritis Crohn’s disease	Phase III (NCT01458951) Phase IV (NCT02092467) Phase II (NCT01470599)
	baricitinib	Rheumatoid arthritis	Phase IV (NCT05660655)
JAK1	upadacitinib	Rheumatoid arthritis Crohn’s disease	Phase III (NCT03086343) Phase III (NCT03345836
	ruxolitinib	Myelofibrosis	Phase II (NCT01340651)
	filgotinib	Crohn’s disease	Phase III (NCT02914600)
	itacitinib	HCC	Phase I (NCT04358185)
STAT	OPB-31,121	HCC	Phase I (NCT01406574)
	IMX-110	Pancreatic cancer	Phase I (NCT03382340)

IL-6 effects can be blocked by a set of antibodies and small molecules, including clazakizumab, sirukumab, olokizumab, and PF-04236921. Clazakizumab and siltuximab directly target the site I of IL-6 for interfering the formation of IL-6 and IL-6R complex, and have been approved for the effectiveness in the treatment of rheumatoid arthritis (RA) and Castleman’s disease in clinical trials [81, 82]. Olokizumab binds to the site III of IL-6, disrupting the recruitment of high-affinity gp130 receptor with IL-6 and IL-6R complex, is also reported to be effective in treating RA patients [83]. Tocilizumab, a humanized monoclonal antibody, is determined to bind to the IL-6 binding site of both membrane and soluble IL-6R, thus blocking classic, *trans-*, as well as cluster signaling [84, 85]. A series of clinical trials have demonstrated the therapeutic benefit of tocilizumab in chronic inflammatory diseases [86–88]. Indeed, treatment using tocilizumab resulted in the marked reductions of lymphadenopathy and inflammatory parameters in Castleman’s disease [89]. Moreover, fibrosis was also strikingly alleviated after tocilizumab injection in lung complications of systemic sclerosis [90]. Recently, a smart nanosystem of palladium nanoplates loaded with tocilizumab can selectively block IL-6R in the liver, ameliorating anemia with hepcidin production and suppressing cancer progression [91]. Moreover, other IL-6R-blocking antibodies, such as sarilumab and satralizumab, have therapeutic effects on COVID-19, RA, and neuromyelitis optica in phase III studies (Table 1). Soluble gp130Fc, named olamkicept or TJ301, is consists of two soluble human gp130 proteins fused with the Fc of human IgG, and selectively targets the IL-6-sIL-6R complex [79]. As neither IL-6 nor sIL-6R alone can bind to gp130, this sgp130Fc is a selective inhibitor of IL-6 *trans*-signaling without interfering the classic signaling. Olamkicept has exhibited promising results in the clinical trials [73, 92, 93]. For example, a phase IIa open label trial evaluating olamkicept in patients with active inflammatory bowel disease (IBD) showed that over 20% patients treated with olamkicept were completely alleviated in clinical symptoms and inflammatory markers [93]. Sgp130Fc treatment in HFD-fed mice presented reduced macrophage accumulation in the adipose tissue, resulting in the improved insulin resistance [94]. Furthermore, sgp130Fc administration, but not anti-IL-6, is more likely to improve the survival in the mice models of cecal ligation and puncture, abdominal aortic aneurysms, and acute lung injury [95–97]. In addition, in a mouse model that mimics human NASH-driven HCC, treatment of olamkicept can significantly regress NASH and markedly reduce NASH-driven HCC [98].

IL-6 binding to IL-6R and gp130 results in the activation of intercellular signal transducers including the phosphorylation of JAK, Src, and STAT3. Therefore, these intercellular signal transducers inhibitors can lead to a global blockade of IL-6 signaling. The clinical trials with JAK/STAT inhibitors are underway for the therapy of cancers and inflammatory diseases (Fig. 5) [12, 99]. For example, ruxolitinib, targeting both JAK1 and JAK2 kinases for inhibiting their phosphorylation, was first approved by the FDA in 2011 for the treatment of myelofibrosis [100, 101]. Tofacitinib, competing with the adenosine triphosphate binding site of both JAK1 and JAK3 for blocking their phosphorylation, was first approved by the FDA in 2012 for the treatment of RA [102]. As reported in COVID-19 patients, both sgp130Fc and JAK inhibitors ruxolitinib can block IL-6 *trans*-signaling with decreased phosphorylation of JAK1 and STAT1/3, resulting in the inhibition of proinflammatory factors production and the alleviation of liver injury [60]. Ruxolitinib also effectively inhibits the JAK/STAT signaling pathway in HCC cells and significantly reduces their proliferation and colony formation [103]. Importantly, evidences from a set of clinical trials showed that the selective JAK1 inhibitors, such as filgotinib and upadacitinib, are more effective than the pan-JAK inhibitor of tofacitinib in the treatment of Crohn’s disease and ulcerative colitis [104–106]. Data from a phase II trial showed that 47% of the Crohn’s disease patients treated with filgotinib achieved the clinical remission compared with 23% in the tofacitinib group [107]. Itacitinib, another JAK1 selective inhibitor, has been investigated in a phase I study of advanced HCC [108]. OPB-31,121, as a STAT3 antagonist targeting its SH2 region, is an orally bioavailable low-molecular-weight compound, and the clinical trial of this drug was conducted in HCC patients [109].

Although the therapeutic effect of intervening IL-6 signaling can be achieved by various inhibitors, ranging from blocking the IL-6 cytokine or its receptor outside of the cell to targeting the kinases and transcription factors inside of the cell, evidences from animal models and pre-clinical studies have demonstrated that inhibition of IL-6 effects also induced adverse effects, thus limiting its use in the clinics in any case [79]. Blocking IL-6 in IBD resulted in severe adverse effects, such as abdominal pain, rather than just ameliorating intestinal inflammation [110]. This problem may be due to the diverse biological effects of the global inhibition of IL-6, and the selective targeting of IL-6 or its downstream effectors may be more important. To date, as evidences from animal models have demonstrated the primary role of IL-6 *trans*-signaling in inflammatory diseases, olamkicept, selectively targeting sIL-6R but not IL-6 or IL-6R, can effectively discriminate between IL-6 classic and *trans*-signaling, thus may be more beneficial than the global blockade of IL-6 [111, 112]. Hence, olamkicept, or the developing next-generation selective inhibitors of IL-6 *trans-*signaling, are considered to be a safe and effective therapeutic strategy for further clinical studies.

## Conclusions

IL-6 is a well investigated pleiotropic cytokine with three different signal modes. Membrane IL-6R are mainly expressed in hepatocytes, immune cells, and some endothelial cells, leading to the limit of IL-6 classic signaling. However, the gp130 protein is widely expressed in tissues [78], representing the extensive responses to IL-6 via the *trans*-signaling activated by IL-6/sIL-6R/gp130 complex. Recently, accumulated evidences have suggested the more important effect of IL-6 *trans*-signaling on liver regeneration and pathological processes. Selective inhibition of IL-6 *trans*-signaling rather than the global blockade of IL-6 might therefore be more effective in the treatment of liver pathologies. Interestingly, HHV-8 encodes a viral homolog of human IL-6, called viral IL-6 (vIL-6) [113]. vIL-6, in contrast to hIL-6, can directly bind to and activate gp130 without the need of hIL-6R. With the activation of downstream signaling cascades, vIL-6 can further increase the production of endogenous IL-6 and enhance the acute-phase responses [114, 115].

It is also generally accepted that IL-6 is a double-edged sword factor for its differential functions in hepatic regeneration, aging, and chronic liver diseases. Under physiological conditions, IL-6 signal is critical for liver regeneration and the proliferation of hepatocytes as to its pro-regenerative effect. During aging progression, the IL-6 level was gradually increased, which enhances its pro-inflammatory effects and even promotes the development of inflammation-associated liver diseases. However, on the other side, high IL-6 level produced by senescent cells can also lead to the reprogramming of hepatocytes, and performs its protective effect on the development of liver diseases with anti-inflammatory activities. Therefore, in order to more accurately intervene the IL-6-mediated liver diseases or aging, it is of prior importance to understand when, where, and how IL-6 works in the physiological and pathological processes in the liver.

## Data Availability

No datasets were generated or analysed during the current study.

## Abbreviations

ADAM: A Disintegrin and Metalloprotease
DC: Dendritic cell
DEN: Diethylnitrosamine
EC: Endothelial cell
EGF: Epidermal growth factor
EMT: Epithelial-mesenchymal transition
ERK: Extracellular-signal regulated kinase
Gp130: 130-kD glycoprotein
HCC: Hepatocellular carcinoma
HFD: High-fat diet
HGF: Hepatocyte growth factor
ICC: Intrahepatic cholangiocarcinoma
IL-6: Interleukin-6
IL-6R: IL-6 receptor
JAK: Janus kinase
MAPK: Mitogen-activated protein kinase
NASH: Non-alcoholic steatohepatitis
PI3K: Phosphatidylinositol 3-kinase
SAMs: Scar-associated macrophages
SASP: Senescence-associated secretory phenotype
SHP-2: Src homology 2 domain-containing protein tyrosine phosphatase 2
SOCS3: Suppressor of cytokine signaling 3
SRC: Src family kinase
vIL-6: Viral IL-6
YAP: YES-associated protein 1
